# Review of Modern Eschweiler–Clarke Methylation Reaction

**DOI:** 10.3390/molecules30173504

**Published:** 2025-08-27

**Authors:** Xiaoli Zhou, Xue Ni, Xianfu Wu, Lihui Yin

**Affiliations:** National Institutes for Food and Drug Control, Beijing 102629, China; zhouxiaoli@nifdc.org.cn (X.Z.);

**Keywords:** amine, methylation, Eschweiler–Clarke, formaldehyde, formic acid

## Abstract

This paper reviews a substantial amount of research related to the Eschweiler–Clarke methylation reaction, summarizing its mechanism, development, and applications, to present the chemical essence and history of this reaction and its importance and application in modern synthetic chemistry. In particular, this review focuses on advances regarding the Eschweiler–Clarke methylation reaction since 2000. This work will provide researchers with a comprehensive and in-depth perspective of the Eschweiler–Clarke reaction, as well as guidance and inspiration for future research and application.

## 1. Introduction

Methyl-substituted amines have widespread applications in chemicals, pharmaceuticals, and materials. Although methyl is a relatively small alkyl group, introducing it into organic molecules can significantly alter the physical, chemical, and biological activities of compounds. Methyl often plays a crucial role in drugs, a phenomenon referred to as the “magic methyl effect” [1,2,3,4]. Nitrogen methylation is also highly regarded for its role in modulating the biological and pharmacological properties of drug molecules [4]. The nitrogen methylation of biomolecules such as peptides, proteins, and amino acids has garnered particular attention [5,6,7,8,9], as it can enhance the oral bioavailability of cyclic peptides [10]. Due to its ability to regulate biological functions, the methylation of nitrogen atoms is considered one of the most important chemical modifications in the natural sciences [7].

The nitrogen methylation of amines often involves nucleophilic reactions using alkylating agents, such as methyl iodide or methyl *p*-toluenesulfonate, but this practice frequently leads to quaternization of the amine. The Eschweiler–Clarke methylation reaction is another significant N-methylation reaction. It is a reductive amination process in which primary or secondary amines are treated with excess formic acid and formaldehyde to obtain N-methylated products. This reaction also exhibits high efficiency [11]. Research has shown that the Eschweiler–Clarke methylation of the optically active amine in which the nitrogen atom is connected to an asymmetric carbon atom does not reduce the optical activity of the resulting tertiary amine. This suggests that the rate of hydrolysis of the isomerized Schiff base is considerably higher than isomerization back to the Schiff base [11,12]. The reaction stops at the tertiary amine stage and typically does not produce quaternary ammonium salts. This reaction is named after the German chemist Wilhelm Eschweiler and British chemist Hans Thacher Clarke. It was first discovered by Eschweiler [13] and later improved by Clarke, who used excess formic acid to facilitate the reaction and achieve higher yields [14]. The reaction is mild and can efficiently methylate various types of amines, making it highly valuable in organic synthesis and pharmaceutical research. Although the Eschweiler–Clarke reaction has been widely studied and applied, with the continuous progress of science and technology and the emergence of new needs, achieving a deeper understanding of and further optimizing the reaction remain of great significance.

This paper reviews the Eschweiler–Clarke methylation reaction to provide a comprehensive perspective, integrate the existing knowledge, and demonstrate the contributions of different researchers in this field, primarily focusing on advances in the Eschweiler–Clarke methylation reaction since 2000. By reviewing the achievements of previous researchers, the key factors affecting the reaction efficiency and selectivity are revealed, and strategies for improving the reaction performance by changing the reaction conditions or using alternative reagents are discussed, as well as how to reduce the environmental impact through the principle of green chemistry. This work will help researchers better understand the mechanism and applications of, as well as possible improvements in, the Eschweiler–Clarke methylation reaction and will provide guidance and inspiration to promote its broader application.

Section 2 describes the mechanism of the Eschweiler–Clarke methylation reaction, and Section 3 presents the applications of this reaction. Section 4 gives a detailed introduction of the development of the Eschweiler–Clarke methylation reaction, focusing on the substitution of formaldehyde or formic acid. Section 5 summarizes the special cases of the Eschweiler–Clarke methylation reaction.

## 2. Mechanism of the Eschweiler–Clarke Methylation Reaction

**Figure 1:**
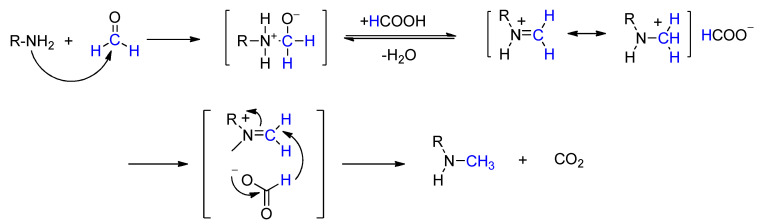
Mechanism of the Eschweiler–Clarke methylation reaction.

Scholars generally believe that, during the Eschweiler–Clarke methylation reaction, the amine condenses with formaldehyde to form a methylene imine (a carbonyl compound–amine condensation reaction). The imine is then protonated by formic acid to form an iminium ion. Subsequently, a hydride ion is transferred from the formate ion to the iminium ion, generating a secondary amine while releasing carbon dioxide (Figure 1). The secondary amine can further condense with a second molecule of formaldehyde to form another iminium ion, which is then reduced by the formate ion to produce a tertiary amine [4]. This mechanism has been demonstrated by using desorption electrospray ionization mass spectrometry to probe the liquid-phase reaction directly [15].

**Figure 2:**
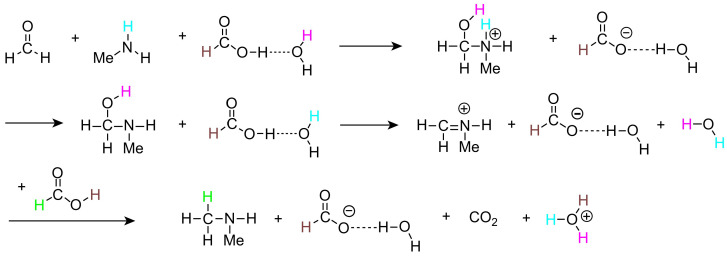
Mechanism of the Eschweiler–Clarke methylation reaction of methylamine, formic acid, formaldehyde, and water molecules.( Different colors show the trajectories of hydrogen atoms)

In 2021, Yamabe et al. studied the hydride transfer process in the Eschweiler–Clarke (amine methylation) reaction involving the system of methylamine, formic acid, formaldehyde, and water molecules by performing density functional theory (DFT) calculations [16]. They employed various DFT methods (including B2PLYP-D3, B3LYP, B3LYP-D, BP86-D, PBE0-D, M06-2X, wB97X-D, and APF-D), as well as the second-order Møller–Plesset perturbation theory (MP2) method, for geometry optimization and calculation of the activation free energies of the transition states. By comparing the results of different DFT methods, the authors found that the M06-2X method was reasonable for tracking this reaction. The DFT calculations suggested that the transition state involving hydride transfer is the rate-determining step. The authors proposed that the reaction involves two molecules of formic acid, where the first molecule assists in the formation of the iminium ion and the second molecule participates in hydride transfer (Figure 2). They found that the size of the water molecule cluster significantly impacts the activation free energy, with an increase in the number of water molecules leading to a decrease in the activation free energy. Their study provided new insights into the Eschweiler–Clarke methylation reaction and its optimization, particularly in terms of considering the impact of water molecule clusters on the reaction.

## 3. Applications of the Typical Eschweiler–Clarke Methylation Reaction

Because N-methylation is an important structural feature of many drug molecules and biologically active molecules, the Eschweiler–Clarke methylation reaction has widespread applications in the field of medicinal chemistry.

**Figure 3:**
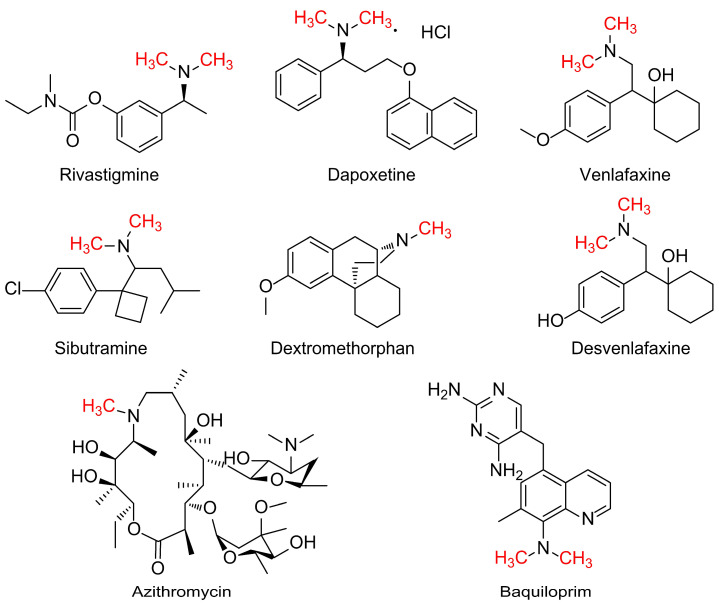
Examples of the classic Eschweiler–Clarke reaction used for the N-methylation of pharmaceutical molecules.

The Eschweiler–Clarke methylation reaction has been used in the synthesis of many drug molecules. For example, it was applied in N,N-dimethylation to prepare Rivastigmine, a centrally selective acetylcholinesterase inhibitor developed by Novartis. This drug was first marketed in Switzerland in 1997 and is used clinically for the treatment of Alzheimer’s disease [17,18,19]. This reaction was also utilized in the N,N-dimethylation of the structure of dapoxetine hydrochloride, a selective serotonin reuptake inhibitor developed by Eli Lilly and Company. This drug was marketed in Europe in 2009 under the brand name Priligy and is used to treat premature ejaculation in males [20,21,22]. It is also employed in the N,N-dimethylation of the structures of venlafaxine and desvenlafaxine. Venlafaxine, developed by the American company Wyeth-Ayerst, was the first selective serotonin and norepinephrine dual reuptake inhibitor antidepressant in the world and was approved by the U.S. Food and Drug Administration (FDA) in 1993 [23,24,25]. Desvenlafaxine, the active metabolite of venlafaxine, was approved by the FDA in February 2008 under the brand name Pristiq and is used for the treatment of major depressive disorder [26]. It is also utilized in the N,N-dimethylation of the structure of sibutramine hydrochloride. Sibutramine hydrochloride is a serotonin and norepinephrine reuptake inhibitor that increases the concentrations of these neurotransmitters in the brain by inhibiting their reuptake, thereby reducing caloric intake and enabling weight loss. This drug was developed by the German company Knoll and was first marketed in the United States in April 1998 under the brand name Meridia for the treatment of obesity [27,28]. The Eschweiler–Clarke methylation reaction is also employed in the N-methylation of the structure of dextromethorphan, a central antitussive drug that primarily works by inhibiting the cough center in the medulla oblongata of the brainstem, thereby blocking the excitation of the vagus nerve [29]. Additionally, the Eschweiler–Clarke methylation reaction is used in the N-methylation of the macrolide antibiotic azithromycin, which was developed by the Croatian pharmaceutical company Pliva in the late 1970s. In 1981, US company Pfizer obtained the patent rights and began selling this drug worldwide [30,31]. Furthermore, the Eschweiler–Clarke methylation reaction is utilized in the N,N-dimethylation of the structure of baquiloprim, an antibacterial potentiator used exclusively for animals. Baquiloprim was developed by the Wellcome Foundation and marketed by Pitman-Moore as a part of a combination formulation called Zaquilan, which was first launched in the UK in 1991. Baquiloprim is a broad-spectrum bacteriostatic agent with in vitro antibacterial activity similar to that of trimethoprim and shows synergistic effects when combined with sulfonamides [32]. The structures of these example drugs are shown in Figure 3.

**Figure 4:**
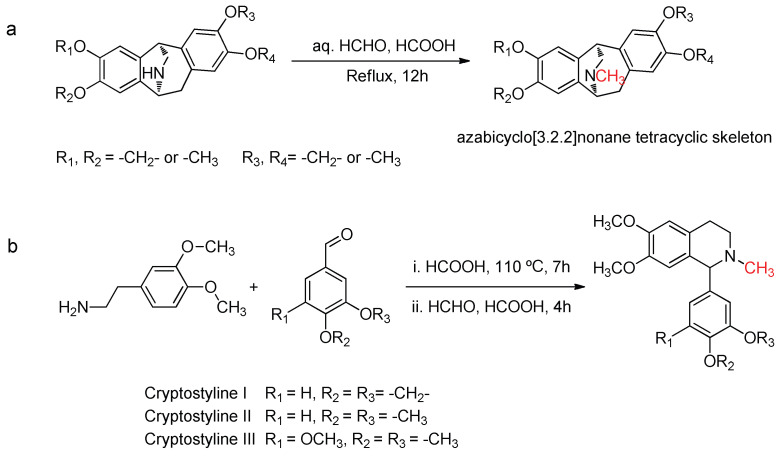
Synthesis of isopavine alkaloids and cryptostylines I, II, and III. (**a**) Final methylation step of isopavine alkaloids. (**b**) One-pot synthesis of cryptostylines I–III.

**Figure 5:**
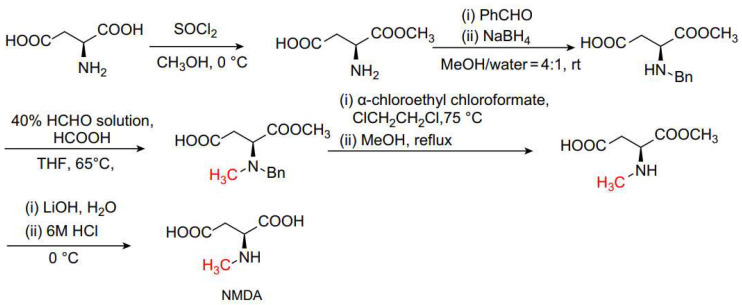
Synthesis of NMDA.

The Eschweiler–Clarke methylation reaction is also commonly used in the synthesis of alkaloids. For example, Pu et al. reported the synthesis of the alkaloid Isopavine, which is primarily isolated from plants in the Papaveraceae and Ranunculaceae families and features a unique azabicyclo[3.2.2]nonane tetracyclic skeleton [33]. The authors utilized the Eschweiler–Clarke methylation reaction for the final methylation step, achieving yields of 72–81%, as shown in Figure 4a. Ruchirawat et al. synthesized a rare class of 1-aryl-tetrahydroisoquinoline alkaloids known as cryptostylines I, II, and III, which were isolated from the plants Cryptostylis fulvaand and Cryptostylis erythroglosa [34]. They achieved one-pot synthesis of cryptostylines I, II, and III by combining the Pictet–Spengler reaction with the Eschweiler–Clarke methylation reaction, with yields of 61–79%, as shown in Figure 4b. Amino acids and sugars are important biomolecules within organisms, playing crucial roles in biological activities. Their synthesis and modification are of great significance in the fields of biology and medicine. The Eschweiler–Clarke methylation reaction is useful for the N-methylation of these compounds. For example, N-methyl-D-aspartic acid (NMDA) is an important natural amino acid, isolated from the nervous and endocrine tissues of humans and animals [35,36]. It acts as an agonist for a subtype of glutamate receptors in the central nervous systems of higher animals [37] and has shown activity as a potential therapeutic drug for diabetes, Parkinson’s disease, and Alzheimer’s disease [38,39]. Xi et al. reported a route for the preparation of NMDA utilizing the Eschweiler–Clarke methylation reaction [40], as shown in Figure 5. This method is convenient for purification, avoids the use of toxic methylating agents, and achieves an overall yield of up to 70%, making it suitable for large-scale production. The Eschweiler–Clarke methylation step has a product yield of 90%, and the product can be employed directly in subsequent reactions without the need for purification.

**Figure 6:**
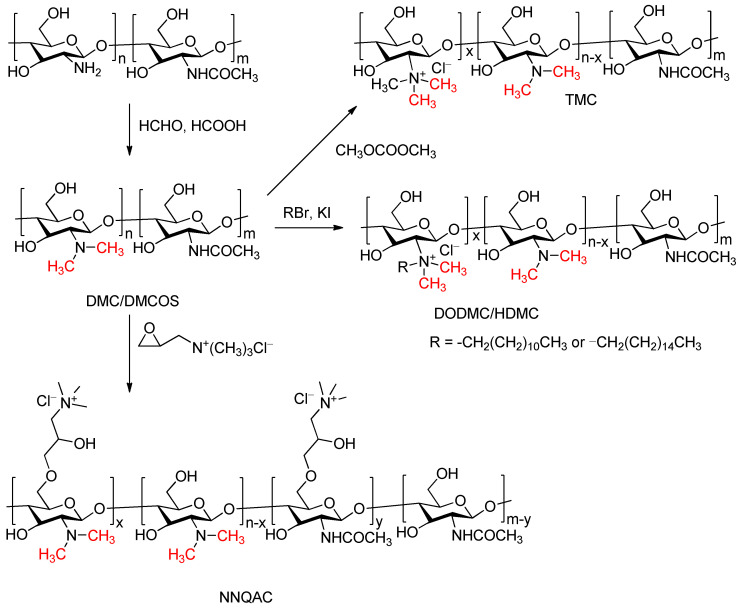
Methylation modification of the chitosan amino group.

The Eschweiler–Clarke methylation reaction is used for the methylation modification of the amino groups in chitosan. Chitosan is a natural polymer obtained by the deacetylation of chitin and has advantages such as being non-toxic, biocompatible, and biodegradable, making it widely applicable in the field of biomaterials. Chemical modification of chitosan is carried out to improve its water solubility and bioactivity. Long et al. obtained N,N-dimethyl chitosan (DMC) through the Eschweiler–Clarke methylation reaction and then performed quaternization using the quaternizing agent dimethyl carbonate to produce N,N,N-trimethyl chitosanium, which has better water solubility and can be widely utilized in drug synthesis, carriers, antibacterial agents, food preservation, and other fields [41]. Li et al. introduced a method of synthesizing DMC via the Eschweiler–Clarke methylation reaction, followed by Hofmann alkylation with bromoalkane to prepare N-dodecyl-N,N-dimethyl chitosan quaternary ammonium salt (DODMC) and N-hexadecyl-N,N-dimethyl chitosan quaternary ammonium salt (HDMC) [42]. The synthesized quaternary ammonium salts exhibited good antibacterial activity, with superior activity against the Gram-positive bacteria *Staphylococcus aureus* compared to that against the Gram-negative bacteria *Escherichia coli*, and the antibacterial activity increased with the length of the alkyl chain. Cao et al. reported a method for the synthesis of N,N-dimethyl-O-quaternary ammonium chitosan (NNQAC) starting from chitosan, involving N,N-dimethylation followed by quaternization with 2,3-epoxypropyltrimethylammonium chloride, which exhibited antibacterial activity against Bacillus subtilis, *E. coli*, and *S. aureus* that was stronger than that of chitosan [43]. Dong et al. reported the synthesis of N,N-dimethyl chitosan oligosaccharides (DMCOSs) through the Eschweiler–Clarke methylation reaction using oligosaccharides. DMCOSs showed enhanced antibacterial activity against four bacterial strains (*S. aureus*, methicillin-resistant *S. aureus*, *E. coli*, and Pseudomonas aeruginosa) and three yeast strains (*Candida albicans*, *Candida tropicalis*, and *Candida parapsilosis*) compared to that of oligosaccharides [44]. The synthetic routes of these compounds are depicted in Figure 6.

**Figure 7:**
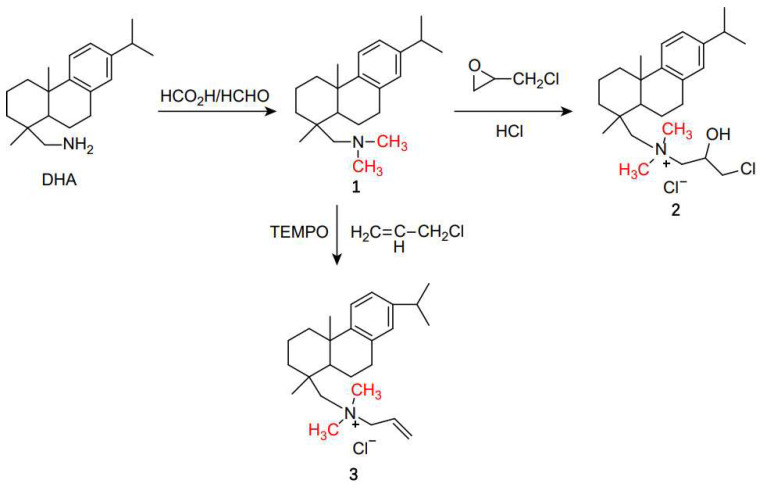
Synthesis of Compounds **2** and **3**.

**Figure 8:**
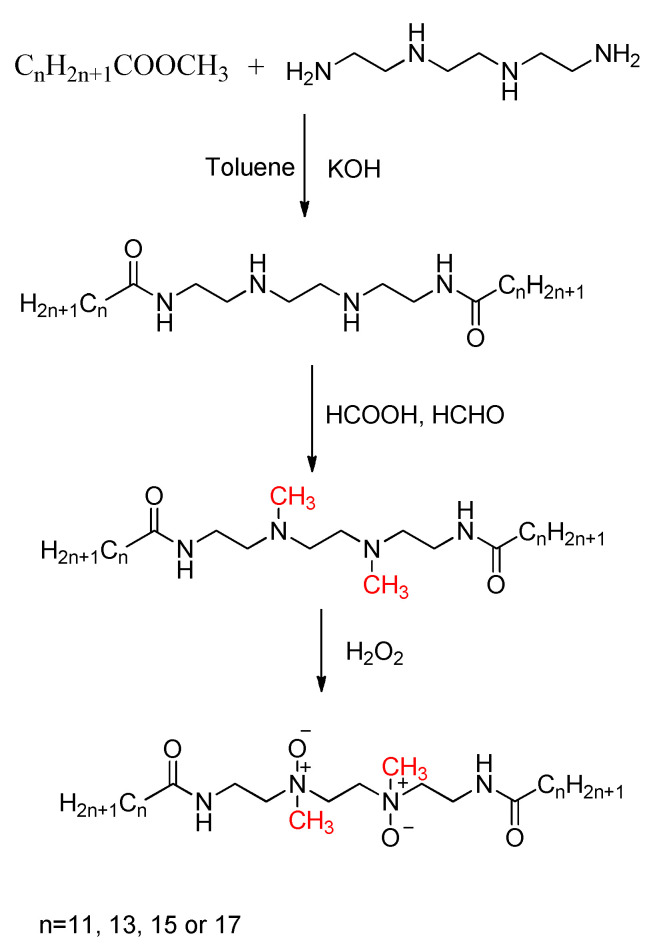
Synthesis of amine oxide gemini surfactants.

The Eschweiler–Clarke methylation reaction has broad applications in the chemical industry. Sudarma et al. proposed a one-pot method of synthesizing 4-allyl-2-(dimethylamino)-6-methoxyphenol from nitro-eugenol, zinc powder, formic acid, and formaldehyde [45]. Zinc powder was used to convert the nitro group into an amine, which then reacted with the Eschweiler–Clarke reagents. The reaction was carried out at 65 °C for 4.5 h in ethanol, with a 59% yield of the target product. Higgins and Winkler synthesized the chiral diamine (1R,6R)-2,7-dimethyl-2,7-diazabicyclo[4.4.1]undecane using the Eschweiler–Clarke methylation reaction, which can serve as a ligand for palladium chloride in asymmetric catalysis to achieve asymmetric induction in Tsuji−Trost alkylation [46]. Ren and Chen presented an environmentally friendly halogen-free cationic antistatic agent and its preparation method, in which long-chain alkyl primary amines (with alkyl chains of C8–C18) react with formic acid and formaldehyde at 50–100 °C for 5–25 h to obtain a tertiary amine, which is then reacted with dimethyl sulfate or diethyl sulfate at 50–100 °C for 1–10 h to prepare the cationic antistatic agent [47]. The product has stable and effective antistatic properties, the raw materials are easy to obtain, and the process is simple, temperature-resistant, low-cost, and convenient to perform, making the cationic antistatic agent widely applicable in coatings, plastics, and fibers. Zhu et al. and Pei et al. developed a novel surfactant using dehydroabietylamine (DHA) as a raw material [48,49]. DHA can be converted into N,N-dimethyl dehydroabietylamine (**1**) via the Eschweiler–Clarke methylation reaction, followed by reaction with epichlorohydrin in the presence of hydrochloric acid to prepare dehydroabietyl-containing active quaternary ammonium salt (**2**). Additionally, in the presence of tetramethylpiperidine-N-oxyl (TEMPO), DMDHA reacts with allyl chloride in the solvent acetonitrile to produce allyl dimethyl dehydroabietyl ammonium chloride (**3**) [50]. The synthetic route is shown in Figure 7. Liu et al. reported the synthesis of a series of amine oxide gemini surfactants containing amide groups [51]. In this process, the raw materials—triethylenediamine and fatty acid methyl esters (including methyl laurate, methyl stearate, methyl palmitate, and methyl oleate)—are reacted under basic conditions in toluene to form N,N’-bis(2-alkylamideethylmethyl)ethylenediamine. Subsequently, the Eschweiler–Clarke methylation reaction is utilized for the reductive methylation of the amine. Oxidation with hydrogen peroxide then yields the amine oxide gemini surfactant, as shown in Figure 8. These amine oxide gemini surfactants exhibit excellent surface activity and good solubility in toluene, and they have potential application in the development of environmentally friendly materials and functional organic materials.

The Eschweiler–Clarke methylation reaction is also commonly applied in the field of polymer materials.

**Figure 9:**
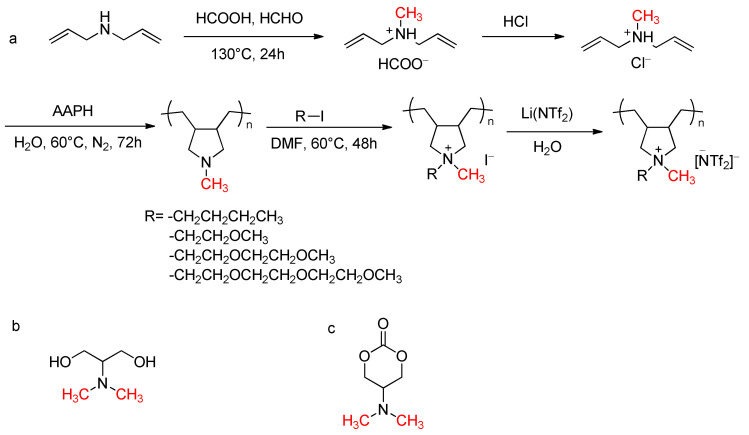
Examples of the classic Eschweiler–Clarke reaction used in polymer materials. (**a**) Synthesis of a novel class of pyrrolidinium-based polyionic liquid electrolytes. (**b**) dimethylamino-1,3-propanediol. (**c**) 5-dimethylamino-1,3-dioxane-2-one.

Dobbelin et al. reported the synthesis of a novel class of pyrrolidinium-based polyionic liquid electrolytes [52]. The synthesis begins with the preparation of diallylmethylamine hydrochloride monomer through the Eschweiler–Clarke methylation reaction of diallylamine. Then, using 2,2′-azobis(2-methylpropionamidine) dihydrochloride (AAPH) as the initiator, the monomer undergoes free radical polymerization in an aqueous medium to form poly(diallylmethylamine hydrochloride). The polymer is subsequently neutralized by adding sodium hydroxide solution in an ice bath, followed by dissolution in DMF and slow addition of the corresponding iodoalkane to perform the quaternization reaction, as shown in Figure 9a. This material exhibits excellent thermal stability and good ionic conductivity, making it suitable for use in a wide range of electrochemical devices.

Wang et al. presented a type of zwitterionic waterborne polyurethane with anti-protein and antimicrobial adhesion properties [53]. The preparation method first involves synthesizing the monomer dimethylamino-1,3-propanediol, which contains both hydroxyl groups and tertiary amino groups, via the Eschweiler–Clarke methylation reaction, as shown in Figure 9b. Dimethylamino-1,3-propanediol is then introduced into the main chain of waterborne polyurethane through an addition polymerization reaction to obtain a polyurethane with tertiary amino groups. The product is further reacted with ester compounds to produce zwitterionic polyurethane, which is finally neutralized with a neutralizing agent and emulsified in water to obtain the zwitterionic waterborne polyurethane. The presence of zwitterions imparts self-emulsifying properties and anti-protein adsorption performance to the polyurethane material, preventing biofouling formation. In biomedical applications, the presence of zwitterions can enhance the biocompatibility of polyurethane materials and reduce the adhesion of proteins and platelets, thereby providing the coating with anticoagulant properties. This zwitterionic polyurethane shows promising application prospects in waterborne coatings for leather and biomedical coatings.

Dong et al. developed a method of preparing 5-dimethylamino-1,3-dioxane-2-one [54]. Starting with serine, the intermediate 2-dimethylamino-1,3-propanediol is obtained via the Eschweiler–Clarke methylation reaction, followed by a cyclization reaction to produce 5-dimethylamino-1,3-dioxane-2-one, as shown in Figure 9c. This compound can be further copolymerized with cyclic carbonate monomers and lactones to prepare antibacterial biomaterials.

**Figure 10:**
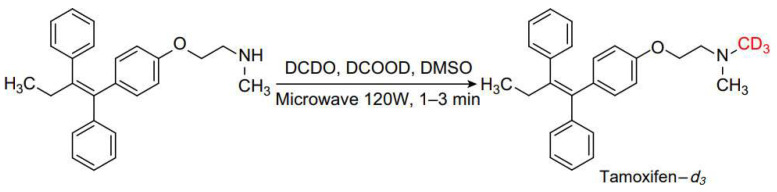
Synthesis of tamoxifen-*d*_3_.

The Eschweiler–Clarke methylation reaction can also be used to introduce isotopes into the nitrogen methyl group of molecules. Tarpey et al. demonstrated that the carbon source of the methyl group attached to the nitrogen atom of the substrate originates from formaldehyde by carbon-14 labeling of the N-methylation of pethidine precursors [55]. Lindeke et al. introduced deuterium into the N-methyl group using the Eschweiler–Clarke methylation reaction, synthesizing three labeled trimethylamines, which were then utilized to prepare labeled choline and acetylcholine [56]. These labeled variants serve as internal standards and tracers in mass spectrometry analysis. Al-Haded et al. reported the synthesis of N-monodeuteriomethyl-2-substituted piperidines using deuterated formic acid and non-deuterated formaldehyde, which were employed to study long-lived nuclear spin states in liquid nuclear magnetic resonance [57]. In 2002, Harding et al. reported a method for isotope labeling using the Eschweiler–Clarke methylation reaction under microwave conditions [58]. They successfully methylated desmethyl tamoxifen to produce tamoxifen-*d*_3_, as shown in Figure 10. Tamoxifen is a widely utilized drug for cancer treatment, and its labeled form has various applications in metabolism and pharmacokinetic studies. This reaction was completed in just 1 min under 120 W microwave conditions, with a yield of 93% for the N-methylation product. The authors used deuterium labeling, but the reaction did not require an excess of reagents, utilizing equimolar amounts of formaldehyde (37% aqueous solution), formic acid, and amine, which is particularly advantageous for radiochemical work.

## 4. Development of the Eschweiler–Clarke Methylation Reaction

To enhance the practicality and environmental friendliness of the Eschweiler–Clarke methylation reaction and to expand its range of applications, researchers have been seeking improved methods of increasing its efficiency, selectivity, and applicability. This section provides a detailed introduction to improvements related to the Eschweiler–Clarke methylation reaction, focusing on the substitution of formaldehyde or formic acid.

### 4.1. Eschweiler–Clarke Methylation Reaction with Formic Acid Substitutes

**Figure 11:**
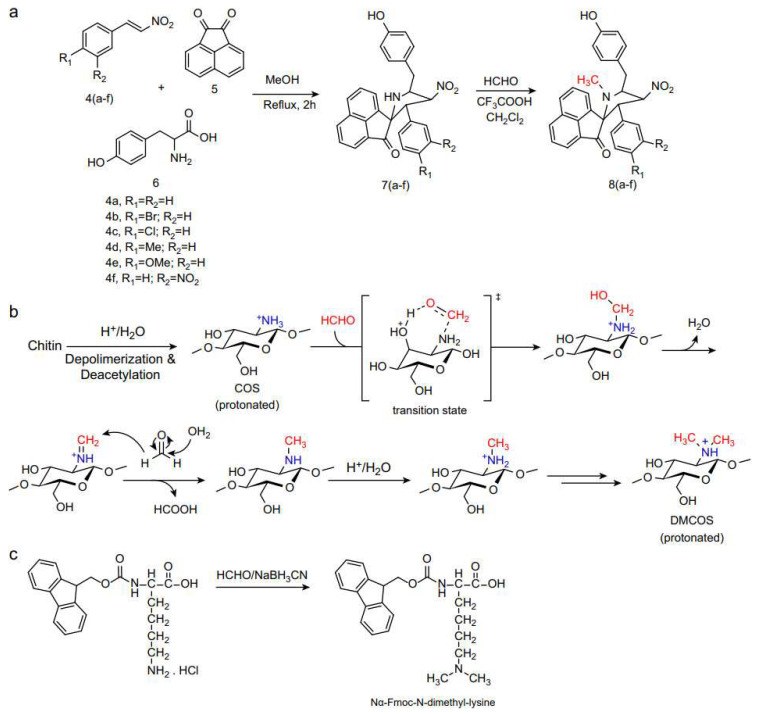
Eschweiler–Clarke methylation reaction with formic acid substitutes. (**a**) Synthetic route to N-methyl spiro-pyrrolidine derivatives. (**b**) one-pot method for the direct synthesis of DMCOSs. (**c**) Synthesis of Nα-Fmoc-NƐ-dimethyl-lysine module using formaldehyde and NaBH_3_CN.

Tirumalai et al. studied the effects of the formaldehyde concentration, pH, and contact time on the formation of N,N-dimethylamphetamine during the reaction of amphetamine with formalin [59]. Under alkaline conditions (pH 9.5) and a high formaldehyde concentration, the decomposition of amphetamine was the most significant, with approximately 90% of amphetamine decomposing on the first day. Under neutral conditions (pH 7.0), a significant amount of N-methylamphetamine was also produced, but to a lesser extent than under alkaline conditions. However, under acidic conditions (pH 3.5), methylation did not occur regardless of the formalin concentration and reaction time. Man et al. reported that, in the presence of a base, primary and secondary aromatic amines could be methylated using formaldehyde as the carbon source and reducing agent [60]. Formaldehyde hydrates with water molecules to form the diol methanediol. Under alkaline conditions, methanediol undergoes proton exchange to change into formic acid, thereby initiating the classic Eschweiler–Clarke methylation reaction, enabling the preparation of tertiary amines from secondary and primary amines. The authors used N-methylaniline with 10 equivalents of formaldehyde (37% *w*/*w*) and 0.2 equivalents of K_2_CO_3_ in toluene at 130 °C for 20 h to obtain N,N-dimethylaniline, with a conversion rate of over 99% and a yield of 74%. They optimized the reaction conditions using N-methylaniline as a standard substrate and found that K_2_CO_3_ and Et_3_N were suitable bases. The reaction showed considerably low yields for amines with large steric hindrance and electron-withdrawing groups; however, it was successful for amines with electron-donating groups. Although the substrate scope is limited, this method demonstrated high efficiency and practicality for certain specific types of amines. Kumar et al. reported the synthesis of a series of novel N-methyl spiro-pyrrolidine derivatives with potential anticancer activity [61,62]. The synthetic route is shown in Figure 11a. Equimolar amounts of Compounds **4**, **5**, and **6** were refluxed in methanol for 2 h, and spiro-pyrrolidine **7** was obtained through a 1,3-dipolar cycloaddition reaction. Spiro-pyrrolidine **7** was then dissolved in CH_2_Cl_2_ and subjected to the Eschweiler–Clarke methylation reaction at room temperature with equimolar amounts of paraformaldehyde and 0.1 molar equivalents of trifluoroacetic acid, yielding the N-methylated spiro-heterocyclic Compound **8**. In this reaction, formaldehyde is oxidized by air to formic acid, which then loses carbon dioxide to serve as a hydride donor [61]. Cheng et al. successfully proposed a convenient one-pot method for the direct synthesis of DMCOSs from chitin through a sequence of acid hydrolysis, depolymerization, deacetylation, and N-methylation reactions [63]. Chitin was reacted with 5% sulfuric acid and 10% formaldehyde at 160 °C for 4 h to produce DMCOSs with a yield of 77%. The product exhibited high degrees of deacetylation, high methylation, and low average molecular weight. The article explored the process of N-methylation under strong acidic conditions and proposed a hydroxyl-assisted mechanism that facilitates the formation of an imine intermediate under strong acidic conditions, which is then reduced to the N-methylated product through a direct hydride transfer process, as shown in Figure 11b.

Rosenau et al. introduced an improved Eschweiler–Clarke methylation reaction method under solvent-free and formaldehyde-free conditions [64]. The authors utilized solid paraformaldehyde and oxalic acid dihydrate as the methylation reagents, avoiding the use of formalin and concentrated formic acid. By replacing formalin and formic acid with paraformaldehyde and oxalic acid and reacting with aliphatic primary and secondary amines at 100–120 °C, the oxalic acid dihydrate decomposes at these temperatures to generate formic acid, which further participates in the reaction. The authors successfully methylated a series of primary and secondary amines, obtaining high-purity products with yields ranging from 84% to 100%. The experiments indicated that the generation of formic acid and the transfer of hydrogen are key steps in the N-methylation process. The reaction does not involve a free radical process, as the addition of antioxidants (such as vitamin E) does not prevent the formation of N-methylated products. This new Eschweiler–Clarke methylation method provides an efficient, safe, and environmentally friendly approach to amine N-methylation, avoiding the use of the harmful chemicals that are employed in traditional methods. Although this method is not suitable for thermally unstable or acid-sensitive amines, it offers an effective methylation strategy for stable amine compounds.

Mulholland et al. reported the synthesis of ^11^C-labeled N-methyl scopolamine, which is considered a candidate for positron emission tomography applications [65]. The authors used [^11^C]formaldehyde to methylate nor-scopolamine under reducing conditions with neutral potassium phosphate. The reaction was conducted in an aqueous solution and completed within 5 min at a temperature of 75–80 °C. The reaction has a high conversion rate. This method is versatile in labeling a variety of [^11^C-methyl]amines. It is fast and efficient, making it highly beneficial in radiochemistry.

Borch and Hassid improved the conditions for the Eschweiler–Clarke methylation reaction by using a formaldehyde–cyanoborohydride system for the methylation of amines [66]. This method is suitable for a wide range of amines and can effectively methylate even those that are particularly difficult to methylate, such as *p*-nitroaniline. Boullais et al. utilized ^11^C-formaldehyde in the presence of NaBH_3_CN as a reducing agent to label ^11^C onto Suriclone at 60 °C in 6 min [67]. Kalle and Anders reported a method for solid-phase reductive methylation of amino groups to synthesize peptides containing N-methyl amino acids [68]. They employed *p*-methoxybenzylamine resin and the traditional Boc/benzyl protection strategy. By mixing the protected peptide resin with an aqueous formaldehyde solution and sodium cyanoborohydride in DMF for 1 h, followed by deprotection/cleavage, N-methyl amino acids were obtained. Twenty common amino acids could be monomethylated directly on the resin, with very low levels of side reactions in most cases. The methylation of lysine in histones is associated with various biological processes, including transcriptional regulation and epigenetic silencing. To study the biological functions of methylation, Huang et al. used formaldehyde and NaBH_3_CN to methylate and construct an N^α^-Fmoc-N^Ɛ^-dimethyl-lysine module, which was utilized for the synthesis of specifically dimethylated and trimethylated peptides of the N-terminal tail of histone H3, with a yield of 93% [69], as depicted in Figure 11c.

Bhattacharyya et al. discovered that using paraformaldehyde, zinc chloride, and zinc borohydride in anhydrous tetrahydrofuran at room temperature for 8–12 h enables the reductive methylation of amines [70]. The article provides a typical experimental procedure for the N-methylation of dibenzylamine under these conditions, with a yield of 90%. By employing zinc chloride and sodium borohydride in dichloromethane at room temperature, the reductive methylation of amines was performed over 8–12 h [71]. In this reaction, zinc chloride acts as a Lewis acid catalyst and a water scavenger, facilitating the formation of the imine intermediate, which is then reduced. Alinezhad et al. reported a method for the reductive methylation of primary and secondary amines to obtain tertiary amines using N-methylpiperidine borohydride as a reducing agent and 37% formaldehyde [72]. The reaction was conducted at room temperature for 6–40 min with a formaldehyde/amine/reduction agent molar ratio of 1:5:1, yielding 80–95% of the product. N-Methylpyrrolidine zinc borohydride can also be used as a reducing agent by optimizing the conditions, and tetrahydrofuran was identified as the best solvent. The optimal conditions were determined to be a 2:1:4 molar ratio of amine/formaldehyde/reduction agent, with the reaction proceeding at 0–10 °C for 10–25 min, with an 88–94% yield [73]. Da Silva et al. reported the reductive methylation of amines and amino acids using zinc and formaldehyde [74]. They proposed that amines could be methylated by treating them with zinc and formaldehyde in an aqueous solution. By simple stirring at room temperature, near-quantitative methylation could be achieved with reaction times ranging from 2 to 20 h, depending on the steric hindrance. Selective monomethylation or dimethylation could be achieved by appropriately choosing the pH, stoichiometry, and reaction time. This method is also applicable to amino acids. Depending on the steric hindrance, high yields and high-purity N-monomethyl amino acids could be obtained within 7 min–2 h by controlling the acidity of the aqueous solution with sodium dihydrogen phosphate. N,N-dimethyl amino acids could also be prepared with longer reaction times or higher reagent excess.

Pearson et al. used Adams’ catalyst (PtO_2_·H_2_O) with formaldehyde as the carbon source and molecular hydrogen as the reducing agent to perform N-methylation in an acidic alcohol/water medium [75]. They synthesized *p*-N,N-dimethylaminobenzoic acid, *p*-N,N-dimethylaminoacetophenone, N,N-dimethylglycine, and N,N-dimethylaniline, with yields of 87%, 70%, 79%, and 74%, respectively.

Guyon et al. reported the methylation of amines using calcium hydride as the hydrogen source, Pd/C as the catalyst, and paraformaldehyde [76]. The reaction was carried out in toluene at 30 °C for 16 h, with the monomethylated product usually being the major product. However, increasing the ratio of calcium hydride and paraformaldehyde led to the formation of dimethylated products.

Natte et al. reported the direct synthesis of functionalized and structurally diverse N-methylamines from nitroarenes and paraformaldehyde, where paraformaldehyde acts as both the methylating agent and reducing agent in the presence of a reusable iron oxide catalyst [77]. This method proceeds without the need for external hydrogen. The versatility of this approach was demonstrated through more than 50 experiments in which important N-methylamines, including bioactive molecules and actual pharmaceuticals, were synthesized. The nitroarenes and paraformaldehyde reacted in 1:1 dimethyl sulfoxide and water in the presence of an iron oxide catalyst and Na_2_CO_3_ at 130 °C for 24–40 h. All nitroarenes were completely converted into the corresponding N,N-dimethylanilines, with yields ranging from 71% to 97%. By controlling the concentration of paraformaldehyde and the reaction time, selective monomethylation could also be achieved, resulting in the synthesis of five selectively monomethylated anilines, with yields between 55% and 81%. Wang et al. reported a method using a Pd/TiO_2_ catalyst for the highly selective synthesis of N-monomethylanilines from nitroarenes and paraformaldehyde through kinetic control [78]. They found that 1,4-dioxane was a suitable solvent for N-monomethylation, whereas the other solvents (ethanol, toluene, cyclohexane, and ethyl acetate) were preferable for N,N-dimethylated products. The reaction was conducted at 60 °C for 24 h, producing N-monomethylated arylamines from various nitroarenes with yields ranging from 72% to 93%. The catalytic scheme was additionally applied to the monomethylation of aniline, achieving a yield of 88%. Interestingly, this method is also suitable for the N-monomethylation of some drugs (nimesulide, clinidipine, 4-aminosalicylic acid, and procaine), with high yields (77–95%).

Rong et al. reported a one-pot method for synthesizing a series of N,N-dimethylanilines using skeletal copper as a catalyst [79]. The skeletal copper catalyst was obtained by leaching aluminum from a Cu-Al alloy by utilizing NaOH solution. This catalyst was then employed to catalyze the N-monomethylation reaction of nitroarenes with formaldehyde under 15 bar of molecular hydrogen at 70–100 °C, with reaction times ranging from 37 to 127 min. The conversion rate of N,N-dimethylaniline derivatives was 100%, with selectivity ranging from 88% to 99%.

Wang et al. successfully developed a CuAlOx catalyst, using paraformaldehyde and hydrogen as the methylation system, to achieve the N-monomethylation of amines [80]. By utilizing tetrahydrofuran as the solvent, aniline and 1.2 equivalents of paraformaldehyde and H_2_ (0.5 MPa) were catalyzed by CuAlOx (with a Cu:Al ratio of 5:5) at a reaction temperature of 120 °C for 9 h. The selectivity for N-methylaniline was 97%, with a yield of 89%. The yields of N-monomethylated substituted anilines ranged from 61% to 85%. Under the same conditions, the yields of secondary amines that were converted into the corresponding N-methylated tertiary amines ranged from 67% to 99%.

### 4.2. Eschweiler–Clarke Methylation Reaction with Formaldehyde Substitutes

Sorribes et al. pointed out that the traditional Eschweiler–Clarke methylation method uses toxic formaldehyde as the carbon source [81]. They established a new method utilizing formic acid as the carbon building block to provide the methyl group in the methylation reaction, with phenylsilane (PhSiH_3_) as the reducing agent to facilitate the methyl transfer from formic acid to amines, achieving methylation. They tested a range of different metal catalysts and found that the Karstedt catalyst ([Pt(CH_2_=CHSiMe_2_)_2_O]) with 1,3-bis(diphenylphosphino)propane as a ligand could catalyze the methylation of amines under mild conditions at room temperature or 60 °C using formic acid and phenylsilane. By employing this method, the authors successfully demonstrated the N-methylation of more than 30 different primary and secondary amines and successfully synthesized five pharmaceutical molecules, including venlafaxine, 4-dimethylaminoantipyrine, imipramine, amitriptyline, and diphenhydramine. The authors further achieved the ability to synthesize [N-^13^C]-labeled drugs, proving the generality and practicality of this catalytic methylation process. They also developed a new heterometallic [Mo_3_Pt(PPh_3_)S_4_Cl_3_(dmen)_3_]BF_4_, which could be used to convert nitroarenes into their N-methylated derivatives using formic acid and phenylsilane [82]. The reaction was carried out in THF at 70 °C for 18 h, producing N,N-dimethylated derivatives with high yields (66–97%).

Zhu et al. reported the methylation of amines using a Pt/C catalyst, with formic acid and hydrosilane as reducing agents [83]. Both primary and secondary anilines could be methylated in the presence of various functional groups, including reducible esters, nitro, and cyano substituents. Aromatic imines could also be reduced and methylated in a cascade manner under the same conditions. The authors found that using PhSiH_3_ as the reducing agent gave the best results, producing N,N-dimethylaniline with a yield of 98% and high selectivity. The N-methylation of primary and secondary aromatic amines and aromatic imines was carried out by utilizing formic acid and phenylsilane in toluene at 80 °C with Pt/C as the catalyst, with a reaction time of 15 h. The heterogeneous reaction produced N-methylated compounds, with yields depending on the substituents, electronic properties, and positions on the benzene ring. The yields of N-methylated products of secondary aromatic amines ranged from 55% to 85%. The yields of N,N-dimethylated products of primary amines ranged from 36% to 95%: *o*-methylthioaniline, *p*-chloroaniline, and *p*-anisidine showed the highest yields of 96%, 96%, and 95%, respectively, and mesidine exhibited the lowest yield of 36%. Most aromatic imines had yields ranging from 72% to 93%.

Singh et al. developed a method for the selective methylation of aromatic amines using a heterogeneous PdAg/Fe_3_O_4_/N-rGO catalyst, with formic acid as the sole methyl source, under additive-free, one-pot reaction conditions [84]. The catalyst facilitated the complete conversion of various substituted aromatic amines to their N-methylated products without aromatic ring hydrogenation, with reaction times of 10–24 h at 140 °C.

Savourey et al. proposed a new method for direct N-methylation that employs formic acid as a unique source of both carbon and hydrogen [85]. Based on a Ru(II) catalyst, the formation of the N-methyl group occurs via an efficient formylation/transfer hydrogenation pathway. Utilizing Ru(COD)(methylallyl)_2_ as the catalyst and triphos (1,1,1-tris(diphenylphosphinomethyl)ethane) as the ligand, the N-methylation of aromatic amines was carried out in the presence of methanesulfonic acid with six equivalents of formic acid. The reaction occurred in THF at 150 °C for 24 h, leading to good conversion to both monomethylated and dimethylated products in most cases. The selectivity between monomethylation and dimethylation of aniline depends on the electronic properties of the substituents on the aryl ring, with strong electron-withdrawing groups favoring monomethylation.

Yu et al. reported a novel catalytic system by utilizing a gold catalyst and formic acid for the reductive conversion of nitro compounds [86]. Using a gold-based solid catalyst and formic acid as the sole reducing agent and hydrogen source, they achieved the direct conversion of nitro compounds into various important amine derivatives, including amines, formamides, benzimidazoles, and dimethylamines. Several nitrobenzenes were successfully converted into their corresponding anilines with yields greater than 99% using an Au/TiO_2_ catalyst (1 mol% gold), formic acid (three equivalents), and toluene as the solvent at 60 °C under a nitrogen atmosphere. When four equivalents of formic acid were used at 70 °C, formylation occurred almost completely (yield >99%). By employing 10 equivalents of formic acid at 140 °C under 40 bar of hydrogen, the only products obtained were N,N-dimethylaniline derivatives (yield >99%).

Qiao et al. reported a method for the copper-catalyzed selective reductive methylation of amines using formic acid as the carbon source and phenylsilane as the reducing agent, enabling the corresponding methylamines to be obtained under mild conditions with good-to-excellent yields [87]. Under the catalysis of copper acetate (Cu(OAc)_2_), formic acid and phenylsilane were employed to methylate various amines at 80 °C for 8 or 16 h. Secondary and primary amines could be N-methylated and N,N-dimethylated, respectively, with yields ranging from 37% to 97%. N-methyl-4-nitroaniline did not react, likely due to the strong electron-withdrawing effect of the nitro group. The authors investigated different metal acetates (Cu(OAc)_2_, Fe(OAc)_2_, Co(OAc)_2_, Ni(OAc)_2_, and Zn(OAc)_2_) using methylaniline as a substrate, and Cu(OAc)_2_ exhibited excellent catalytic activity (conversion: >99%, yield: 99%). They also examined the catalytic effects of other copper salts and observed that Cu(acac)_2_, Cu(OTf)_2_, and CuF_2_ showed good conversion rates (>99%) and yields (76–99%).

Fu et al. discovered a boron-based catalyst (B(C_6_F_5_)_3_) capable of catalyzing the direct alkylation of amines with carboxylic acids utilizing silane as the reducing agent [88]. This method does not require the use of a metal catalyst and exhibits excellent selectivity and functional group compatibility. In di-n-butyl ether, the N-methylation of amines was carried out by employing formic acid and polymethylhydrosiloxane under the catalysis of triphenylborane at a reaction temperature of 100 °C for 8–10 h. N-methylated products were obtained from secondary aromatic amines, whereas N,N-dimethylated products were isolated from primary aromatic amines, with yields ranging from 68% to 97%. DFT calculations and experimental studies revealed that the acid and amine first condense to form an amide through a catalyst-free mechanism, which is then reduced to produce the alkylated product via a mechanism catalyzed by the Lewis acid (B(C_6_F_5_)_3_) [89].

## 5. Special Cases of the Eschweiler–Clarke Methylation Reaction

**Figure 12:**
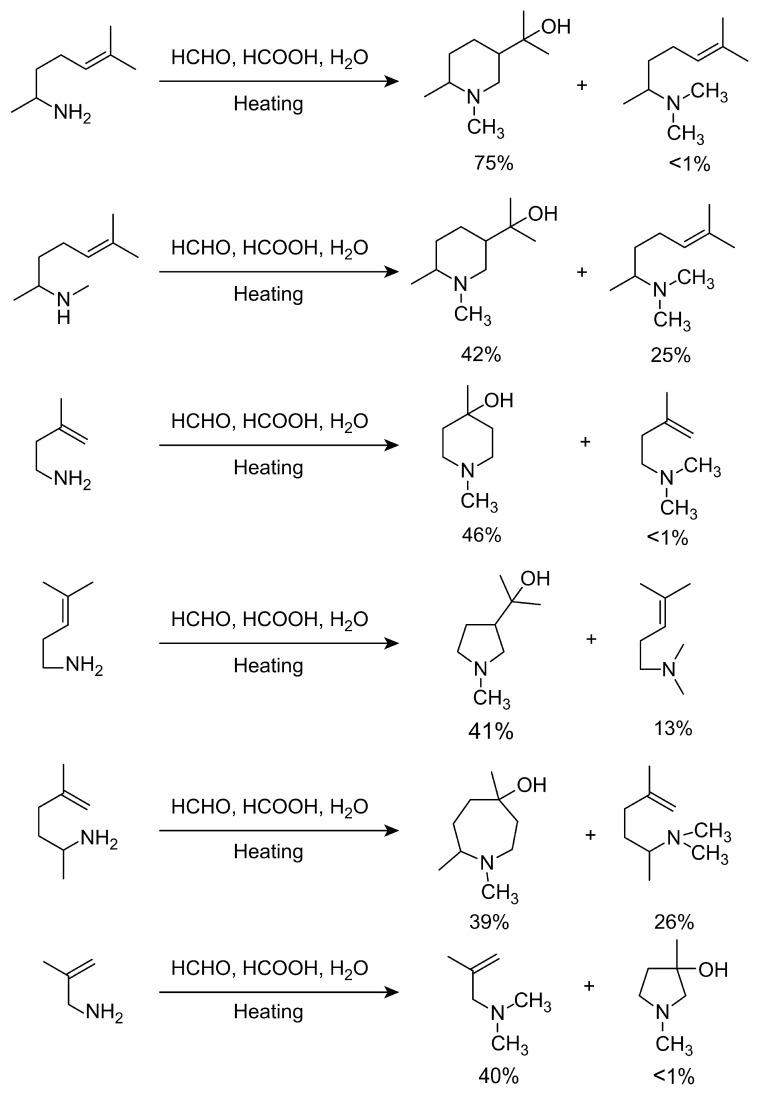
Cyclization of olefinic amines during N-methylation.

During the Eschweiler–Clarke methylation of amines, N-methylated products may not form due to the structure of the substrate, or cyclization may occur during the methylation process. Cope and Burrows treated 1,5-dimethyl-4-hexenylamine with aqueous formaldehyde and formic acid, and instead of the expected dimethylamine forming, a cyclization reaction occurred, resulting in the N-methylated cyclized product piperidine methanol, with a yield of 75% for the isolated cyclized product [90]. As shown in Figure 12, the cyclization mechanism involves the stabilization of the carbocation derived from the iminium ion. However, due to the thermodynamic instability of the carbocation, 1-methyl-4-pentenylamine is converted into the normal N,N-dimethyl derivative rather than a cyclized product. The authors also extensively studied cyclization reactions accompanying the Eschweiler–Clarke methylation of several other alkenylamines and found that 3-methyl-3-butenylamine, 4-methyl-3-pentenylamine, 1,4-dimethyl-4-pentenylamine, and 6,6-dimethyl-2-tetralinylethylamine all produced cyclic products either fully or partially, whereas 2-methylallylamine did not [90]. They concluded that cyclization occurs before the methylation of 1,5-dimethyl-4-hexenylamine.

**Figure 13:**
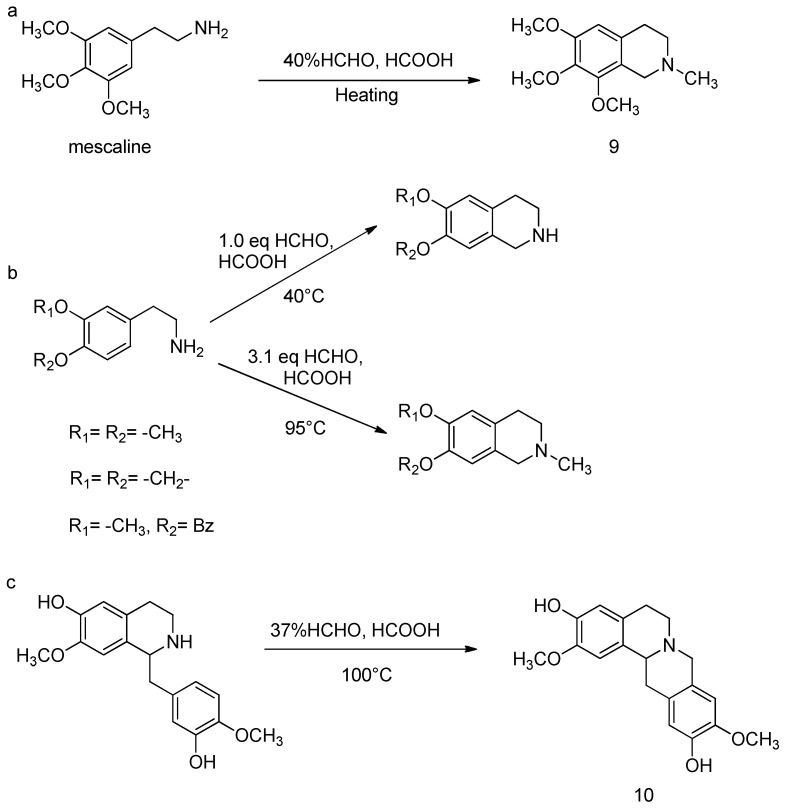
Cyclization of β-phenylethylamine during N-methylation. (**a**) N-methylation of mescaline. (**b**) Tetrahydroisoquinoline alkaloids from arylethylamines and paraformaldehyde in formic acid. (**c**) Eschweiler–Clarke methylation after Pictet–Spengler to give 5,8,13,13a-tetrahydro-2,10-dimethoxy-dibenzo[a,g]quinolizine.

Certain cyclic substituted β-phenylethylamines undergo the Pictet–Spengler reaction during this methylation process, resulting in cyclization to form tetrahydroisoquinolines. Castrillón reported that mescaline, when heated with formic acid and 40% formaldehyde in a steam bath for 12 h or refluxed in an oil bath for 8 h, produced N-methyl-6,7,8-trimethoxy-1,2,3,4-tetrahydroisoquinoline (**9**) with yields of 51.2% and 79.7%, respectively [91], as shown in Figure 13a. Ruchirawat et al. synthesized several simple tetrahydroisoquinoline alkaloids by reacting suitable arylethylamines with paraformaldehyde in formic acid [92], as depicted in Figure 13b. Kametani et al. used 1,2,3,4-tetrahydro-6-hydroxy-1-(3-hydroxy-4-methoxybenzyl)-7-methoxyisoquinoline to synthesize the 5,8,13,13a-Tetrahydro-2,10-dimethoxy-6H-dibenzo[a,g]quinolizine-3,11-diol (**10**) under Eschweiler–Clarke methylation conditions, where the Pictet–Spengler reaction occurred with a yield of 88.45% [93], as illustrated in Figure 13c.

**Figure 14:**
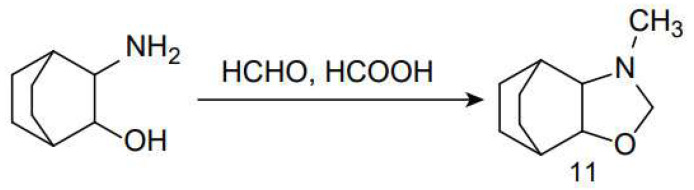
Cyclization of *cis*-3-Aminobicyclo[2.2.2]octan-2-ol.

Nelson found that, during the Eschweiler–Clarke methylation reaction of *cis*-3-aminobicyclo[2.2.2]octan-2-ol, an oxazole structure (**11**) was formed instead of a tertiary amine [94], as depicted in Figure 14.

**Figure 15:**
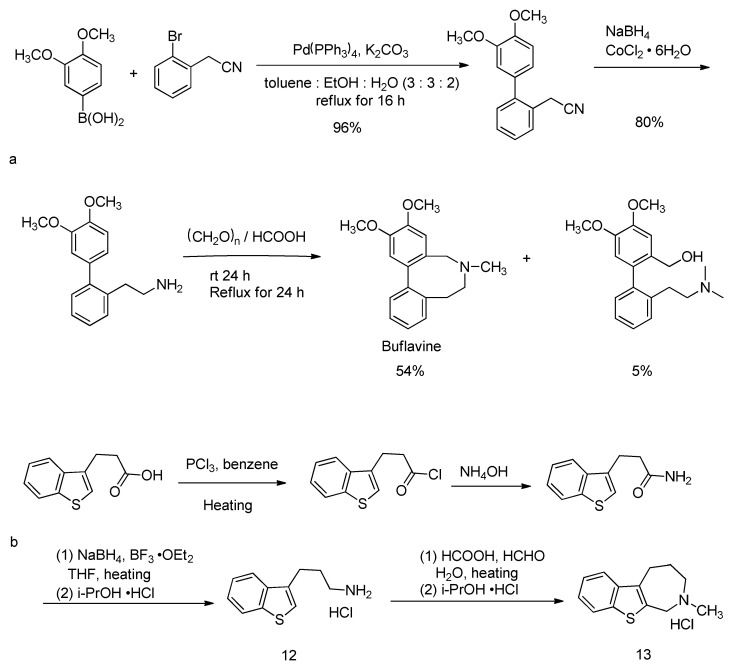
Synthesis of buflavine and 2-methyl-2,3,4,5-tetrahydro-1H-[1]benzothieno[2,3-c]asepine(**13**). (**a**) Synthesis of buflavine. (**b**) Synthesis of 2-methyl-2,3,4,5-tetrahydro-1H-[1]benzothieno[2,3-c]azepine(**13**).

Sahakitpichan and Ruchirawat reported the synthesis of the Amaryllidaceae alkaloid buflavine [95]. The natural product, with potential α-adrenergic release and anti-serotonin activity, was synthesized in three steps through Suzuki–Miyaura cross-coupling, reduction, and the cascade reactions of Pictet–Spengler and Eschweiler–Clarke N-methylation, as illustrated in Figure 15a.

Eresko et al. started with benzothiophene-3-propionamide, which was reduced using diborane to obtain the corresponding amine. The amine was then converted into 3-(1-benzothiophen-3-yl)propylamine hydrochloride (**12**), which finally formed an azepane ring under Eschweiler–Clarke reaction conditions. This reaction was utilized to synthesize 2-methyl-2,3,4,5-tetrahydro-1H-[1]benzothieno[2,3-c]azepine (**13**) [96], as shown in Figure 15b.

**Figure 16:**
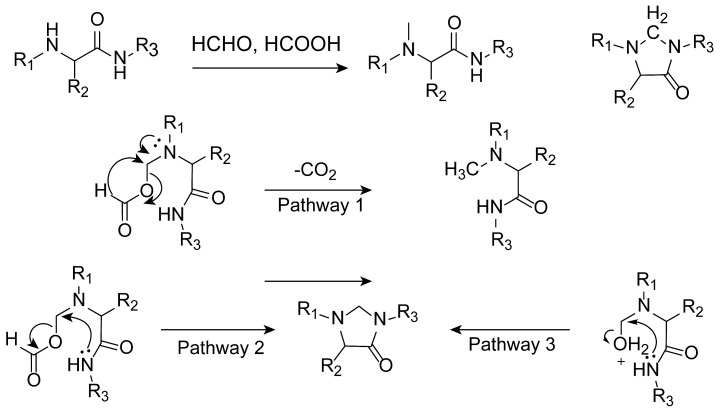
α-Amino amides under Eschweiler–Clarke methylation conditions.

Chen and Sung, during the N-methylation of α-amino amides using the Eschweiler–Clarke methylation reaction, discovered that the main product was not the expected N-methylated product but rather a cyclized product, imidazolidin-4-one [97]. The Eschweiler–Clarke methylation of α-amino amides may involve competition between the decarboxylation of hemiaminals (pathway 1) and the cyclocondensation of hemiaminal analogs (pathways 2 and 3), as shown in Figure 16. The article compared the outcomes of the Eschweiler–Clarke methylation on α-amino acids and α-amino amides. For α-amino acids, the reaction produced N-methylated products. However, for α-amino amides, the reaction typically resulted in cyclocondensation products, specifically imidazolidin-4-ones, rather than N-methylated products. The authors noted that, when all three substituents on the α-amino amide are large, the rates of cyclocondensation and N-methylation become comparable. In such cases, both the cyclocondensation product and the N-methylated product can be obtained.

**Figure 17:**
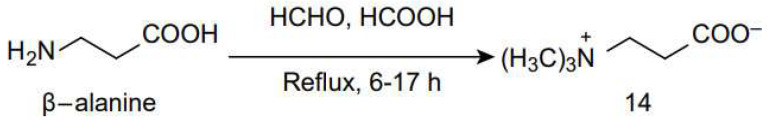
β-alanine under Eschweiler–Clarke methylation reaction conditions.

Rahal and Badache. discovered that, under Eschweiler–Clarke methylation reaction conditions, the reaction of β-alanine with formaldehyde and formic acid unexpectedly produced the corresponding β-zwitterion (**14**) rather than the expected N,N-dimethyl β-alanine, with a yield of 82% [98], as shown in Figure 17.

**Figure 18:**
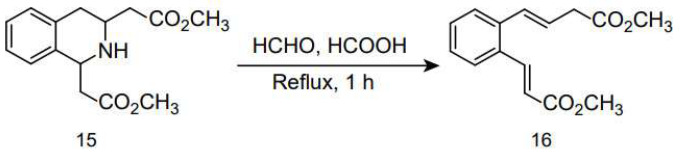
Cyclic *p*-aminodiesters under Eschweiler–Clarke methylation reaction conditions.

Watanabe and Wakabayashi reported a novel deamination cleavage reaction under Eschweiler–Clarke methylation conditions (37% formaldehyde, formic acid, reflux), which converted a cyclic *p*-amino diester (**15**) into methyl (E)-4-[*o*-(2(E)-(methoxycarbonyl)vinyl)phenyl]-3-butenoate (**16**), with a yield of 73% [99], as shown in Figure 18.

**Figure 19:**
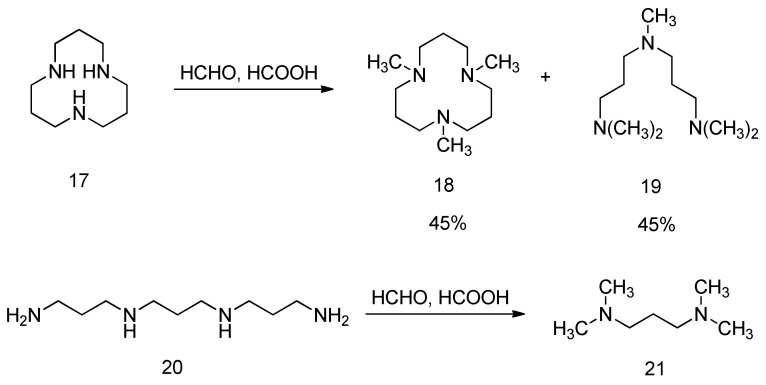
Polyamines are cleaved into the fragments of methylated products during the Eschweiler–Clarke methylation reaction.

Alder et al. discovered that certain polyamines undergo further cleavage during the Eschweiler–Clarke methylation reaction, producing methylated fragments of the original polyamine [100]. For example, when 1,5,9-triazacyclododecane (**17**) is converted into 1,5,9-trimethyl-1,5,9-triazacyclododecane (**18**), 2,6,10-trimethyl-2,6,10-triazaundecane (**19**) is also formed. The methylation product of the acyclic polyamine 1,5,9,13-tetraazatridecane (**20**) is 1,1,3,3-tetramethylpropylenediamine (**21**), as shown in Figure 19. The authors tested a series of linear triamines and tetraamines under Eschweiler–Clarke methylation conditions and found that only polyamines containing the H_2_N(CH_2_)_3_NHR group lead to cleavage, with the cleavage product being 1,1,3,3-tetramethylpropylenediamine.

**Figure 20:**
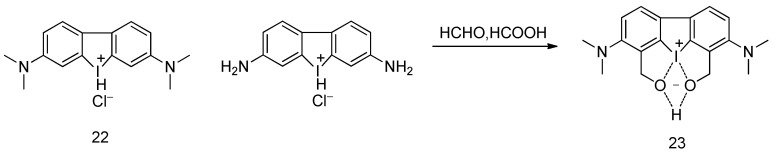
Formation of salt within cyclic iodonium.

Hou et al. attempted to prepare 3,7-di(dimethylamino)dibenzo[b,d]iodinium pentacyclic carboxylate (**22**), a compound with anti-radiation physiological activity, using the Eschweiler–Clarke methylation reaction [101]. However, they unexpectedly obtained a novel cyclic iodonium inner salt, dibenzo[b,d]iodinium inner salt (**23**), with a yield of 73%, as shown in Figure 20.

## 6. Conclusions

Although the methyl group is very small, it has broad and important roles in many fields. The Eschweiler–Clarke methylation reaction is a reductive amination technique with widespread applications in organic synthesis and medicinal chemistry. This paper reviewed the reaction mechanism, development, potential special cases, and numerous practical application examples of this reaction. While the classical Eschweiler–Clarke methylation reaction, employing formic acid and formaldehyde, remains a valuable and widely used tool because of its operational simplicity, mild conditions, and ability to stop at the tertiary amine stage, ongoing research is actively addressing its limitations and expanding its scope. A major research thrust towards modernizing the Eschweiler–Clarke methylation reaction involves the development of catalytic methodologies, particularly metal-catalyzed systems, targeting enhanced efficiency, selectivity, and sustainability. Breakthroughs in non-noble-metal catalysis have stimulated renewed interest in this century-old reaction, propelling it toward a greener upgrade. Inexpensive catalysts enable the efficient N-methylation of amines, nitroarenes, and nitriles under renewable C_1_ sources such as CO_2_, formic acid, or paraformaldehyde, while exhibiting excellent compatibility with sensitive functional groups, that is, halogens, esters, amides, and alkenes. Hence, the limitations of classical Eschweiler–Clarke conditions regarding acid-labile substrates are being overcome [102]. Meanwhile, the cyclization side reactions that once accompanied the Eschweiler–Clarke process are now being deliberately harnessed, with this approach emerging as an additional route towards high-value nitrogen heterocycles. The review conducted by Umar and Luo [103] catalogues a series of “atypical” events occurring under Eschweiler–Clarke conditions. For instance, when the substrate framework possesses nucleophilic or olefinic motifs, the reaction can evolve into a tandem “methylation–cyclization” sequence, and this behavior constitutes a powerful tool for the construction of complex nitrogen-containing scaffolds. With continuous exploration and development, the application scope of this reaction has been greatly expanded, while its environmental friendliness and ease of operation have been enhanced. The Eschweiler–Clarke methylation reaction will continue to play an important role in future chemical synthesis and pharmaceutical research. This review provides a comprehensive view of the Eschweiler–Clarke methylation reaction, which should aid in understanding the reaction and provide insights for its development and application.

## Data Availability

No new data were created or analyzed in this study. Data sharing is not applicable to this article.

## Abbreviations

The following abbreviations are used in this manuscript:

DFT	Density functional theory
DHA	Dehydroabietylamine
DMC	N,N-dimethyl chitosan
DMCOS	N,N-dimethyl chitosan oligosaccharide
DMDHA	N,N-dimethyl dehydroabietyl
NMDA	N-methyl-D-aspartic acid
